# A transcriptomic, proteomic, and functional genetic atlas dissects neurofibromin function in the peripheral nervous system

**DOI:** 10.1073/pnas.2506823122

**Published:** 2025-06-30

**Authors:** Harish N. Vasudevan, Nadia Arang, Maria Sacconi Nunez, Patrick Kennedy, Emily Payne, Sarah Mohabeer, Julian Chien, Aaron Wright, Matthew J. Sale, Nevan J. Krogan, Antoine Forget, Frank McCormick

**Affiliations:** ^a^Department of Radiation Oncology, University of California San Francisco, San Francisco, CA 94143; ^b^Department of Neurological Surgery, University of California San Francisco, San Francisco, CA 94143; ^c^Department of Cellular and Molecular Pharmacology, Quantitative Biosciences Institute, University of California, San Francisco, CA 94158; ^d^Department of Cellular and Molecular Pharmacology, University of California San Francisco, San Francisco, CA 94158; ^e^Department of Cellular and Molecular Pharmacology, Helen Diller Family Comprehensive Cancer Center, University of California San Francisco, San Francisco, CA 94158

**Keywords:** neurofibromin, Ras inhibitor, peripheral nervous system

## Abstract

The *NF1* tumor suppressor gene is recurrently mutated in human cancers and is associated with the neurofibromatosis type 1 (NF-1) cancer predisposition syndrome. *NF1* encodes neurofibromin, a Ras guanosine triphosphate (GTPase) activating protein that negatively regulates Ras signaling. *NF1* mutation accordingly leads to Ras misactivation and downstream activation of RAF/MEK/ERK signaling, leading to the approval of the MEK inhibitor selumetinib for NF-1 associated peripheral nervous system (PNS) tumors. However, how *NF1* loss modifies response to selumetinib and the utility of targeting additional upstream inputs or downstream outputs of Ras these tumors remain unclear. Here, we perform RNA-sequencing, phosphoproteomic, pharmacologic, and proximal proteomic analysis across a panel of CRISPR interference immortalized peripheral nerve (iPN) cells to systematically dissect the function of neurofibromin loss. Small guide *NF1* (sg*NF1*) repression is sufficient to increase Ras GTP levels and alter gene expression to promote cell proliferation and dedifferentiation, with sg*NF1* iPNs showing decreased sensitivity to selumetinib due to altered feedback regulation to Ras/RAF/MEK/ERK. Upstream small guide *PTPN11* (sg*PTPN11*) repression leads to the inverse gene expression signature, decreasing cell proliferation and promoting differentiation, and sg*PTPN11* iPNs are more sensitive to selumetinib. However, upstream sonof sevenless 1 inhibition shows limited efficacy in iPNs due to compensation by SOS2. Finally, proximal proteomics reveals Kirsten rat sarcoma virus (KRAS), but not Harvey rat sarcoma virus (HRAS) or neuroblastoma Ras viral oncogene homolog (NRAS), associates with neurofibromin in iPN cells, and pan-KRAS inhibition is sufficient to block ERK activation and CDK1/2 activation in *NF1* mutant cells, suggesting blocking KRAS may be a therapeutic approach for *NF1* mutant PNS tumors.

## Significance

Personalized medicine based on pharmacologically targeting specific mutations have revolutionized cancer. In contrast to gain of function oncogenes that can be directly inhibited, such approaches are challenging for loss of function tumor suppressors. One such example is the *NF1* tumor suppressor gene encoding neurofibromin, which negatively regulates Ras small GTPases and thus leads to RAF/MEK/ERK activation. Although MEK inhibitors are approved for some *NF1* mutant tumors, better therapies are needed for people who do not respond to or cannot tolerate MEK inhibitors. To identify additional therapeutic strategies for *NF1* mutant tumors, we systematically analyze upstream inputs and downstream outputs to Ras following *NF1* loss. Our data suggest direct KRAS inhibition may be a promising approach for neurofibromatosis type 1.

Loss of the Ras GTPase activating protein (GAP) neurofibromin encoded by the *NF1* gene causes the congenital cancer predisposition syndrome neurofibromatosis type-1 (NF-1) (1). People with NF-1 are at increased risk of peripheral nervous system (PNS) tumors such as plexiform neurofibromas (pNFs) that can transform into malignant peripheral nerve sheath tumors (MPNSTs) (2). *NF1* loss leads to Ras/rapidly accelerated fibrosarcoma (RAF)/mitogen activated protein kinase kinase (MEK)/extracellular signal regulated kinase (ERK) activation (3), and the MEK inhibitor selumetinib is approved for NF-1 associated pNFs (4, 5), yet selumetinib resistance through both Ras dependent and Ras independent mechanisms (6) remains an unmet challenge. Alternate approaches inhibiting upstream inputs to Ras such as the guanine nucleotide exchange factor (GEF) son of sevenless 1 (SOS1) or the SH2 domain-containing tyrosine phosphatase-2 (SHP2) encoded by the protein tyrosine phosphatase nonreceptor type 11 (*PTPN11*) gene have shown preclinical efficacy (7–9). Despite the promise of MEK and upstream SOS1/SHP2 inhibition for NF1-associated tumors (10), neither approach targets direct neurofibromin substrates, and it is unclear how selective *NF1* loss affects responses to these agents, a critical consideration for maximizing the therapeutic window in people with *NF1^−/−^* tumors arising in an *NF1^+/−^* heterozygous germline background. More broadly, a systematic comparison of how perturbing *NF1* or upstream inputs to Ras such as SOS or SHP2 affects downstream effector activation in the PNS has not been conducted, and the full complement of NF1 interactors and pharmacologic dependencies in PNS cells remains incompletely understood. To address this knowledge gap, we combine CRISPR interference (CRISPRi) based functional genetic perturbations with proteomic, transcriptomic, and pharmacologic approaches to define the effects of *NF1* loss on regulating upstream inputs to or downstream outputs from Ras.

## Results

**Fig. 1.**
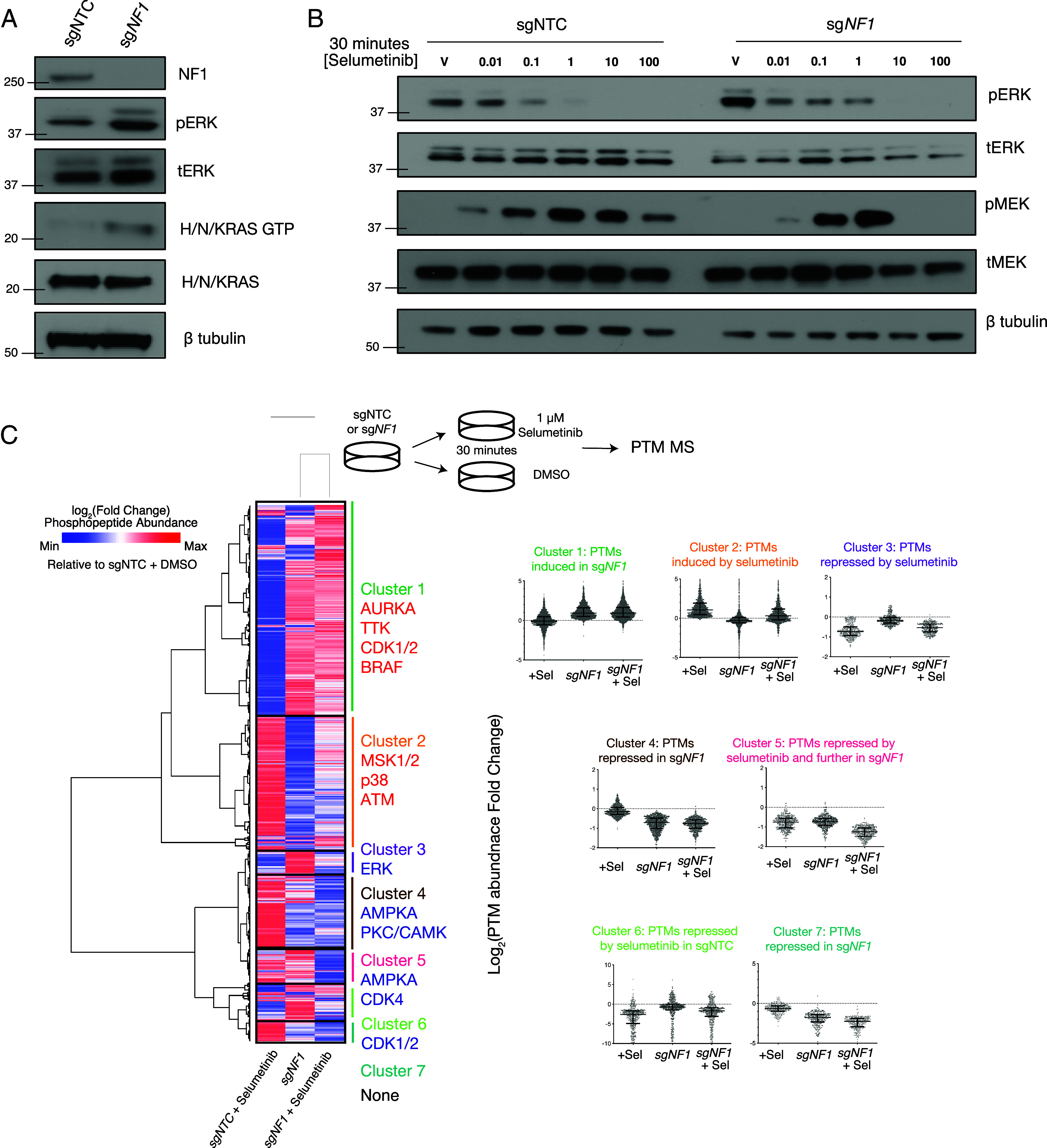
CRISPRi sg*NF1* repression in iPN cells decreases selumetinib sensitivity due to altered feedback regulation at the transcriptional and phosphoproteomic levels. (*A*) CRISPRi sg*NF1* iPN cells show increased pERK and Ras GTP levels. (*B*) Selumetinib dose–response reveals sg*NF1* iPNs exhibit decreased biochemical response compared to sgNTC iPNs. (*C*) Phosphoproteomic mass spectrometry (PTM-MS) of sgNTC or sg*NF1* iPNs treated with 30 min 1 μM selumetinib identifies seven PTM clusters. Clusters 1 and 4 are regulated by sg*NF1* repression alone, clusters 2 and 3 are regulated by selumetinib regardless of *NF1* repression, and the remaining clusters 5, 6, and 7 are regulated based on a combination of selumetinib treatment and *NF1* repression.

To investigate neurofibromin function in the PNS, we used the CRISPRi system to repress *NF1* in immortalized peripheral nerve (iPN) cells, which was validated by qRT-PCR (*SI Appendix*, Fig. S1*A*). Immunoblotting revealed sg*NF1* repression was sufficient to increase pERK and H/N/K Ras GTP levels (Fig. 1*A*), and CRISPRi sg*NF1* cells demonstrated significantly increased growth compared to small guide non targeting control (sgNTC) controls (*SI Appendix*, Fig. S1*B*). Biochemical analysis of dose–response to the MEK inhibitor selumetinib showed sg*NF1* iPNs were less sensitive to selumetinib as measured by phospho-ERK with altered feedback rebound of phospho-MEK compared to sgNTC controls (Fig. 1*B* and *SI Appendix*, Fig. S1*C*). To understand the transcriptomic effect of sg*NF1* repression, we next performed RNA-sequencing of sg*NF1* compared to sgNTC iPNs (*SI Appendix*, Fig. S2*A* and Dataset S1). CRISPRi sg*NF1* iPNs induced genes were enriched for positive regulation of cell proliferation, consistent with their increased cell growth, and negative regulation of cell differentiation (*ID2, SNAI2, TWIST2*) (*SI Appendix*, Fig. S2*B*). From a signaling perspective, sg*NF1* iPNs induced genes that are negative regulators of phosphorylation (*DUSP4, DUSP6*) and repressed genes enriched for classic receptor tyrosine kinase (RKT) signaling feedback regulators such as RNA binding and kinase binding genes (*SI Appendix*, Fig. S2*B*).

To better define the signaling networks altered in sg*NF1* iPNs and selumetinib treatment, we performed Phosphoproteomicmass spectrometry (PTM-MS) on control sgNTC or sg*NF1* iPNs treated with 1 μM selumetinib or vehicle dimethyl sulfoxide (DMSO) control for 30 min (Fig. 1*C*). Pairwise differential phosphopeptide analysis revealed sg*NF1* iPNs were associated with significantly increased activity of the cell cycle kinases CDK1 and CDK2 (*SI Appendix*, Fig. S3*A*) consistent with the observed increase in cell growth (*SI Appendix*, Fig. S1*B*) and RNA-sequencing data (*SI Appendix*, Fig. S2). Control sgNTC iPNs treated with selumetinib demonstrated significant repression of MEK1 and MEK2 kinase activity (*SI Appendix*, Fig. S3*B*) consistent with our selumetinib dose–response (Fig. 1*B*). In contrast, sg*NF1* iPNs treated with selumetinib did not show significant repression of MEK1 or MEK2 (*SI Appendix*, Fig. S3*C*). To more globally define patterns of kinase activity across genetic *NF1* repression and selumetinib treatment, we performed hierarchical clustering of our three experimental conditions using the log fold change of differentially regulated phosphopeptides relative to sgNTC iPNs treated with DMSO. We identified a total of seven significantly regulated phosphopeptide clusters (Fig. 1*C* and Datasets S2 and S3). Cluster 1 contained phosphopeptides induced by sg*NF1* repression and not regulated by selumetinib, showing enrichment for kinase activity of AURKA, TTK, CDK1/2, and BRAF consistent with the observed differences in cell growth (*SI Appendix*, Fig. S1*B*), proliferation gene expression signatures (*SI Appendix*, Fig. S2*B*), and immunoblots of increased RAS/RAF activity (Fig. 1*A*). Cluster 4 contained phosphopeptides repressed by sg*NF1* repression and not regulated by selumetinib, showing enrichment for AMPKA and PKC/CAMK activity. Clusters 2 and 3 represented phosphopeptides induced or repressed by selumetinib, respectively, in both sgNTC and sg*NF1* cells; consistent with MEK inhibitor treatment, the stress activated kinases MSK1/2 were induced by selumetinib while ERK kinase activity was repressed by selumetinib. Cluster 6 contained phosphopeptides repressed by selumetinib in sgNTC but not sg*NF1* cells and were enriched for CDK1/2 activity, consistent with a differential effect of selumetinib on sgNTC *NF1* intact compared to sg*NF1* deficient iPNs. Finally, Clusters 5 and 7 were notable for PTMs repressed in sg*NF1* iPNs and further repressed upon selumetinib treatment, showing enrichment for AMPKA and CDK4 kinase activity. Taken together, these data reveal that sg*NF1* repression activates RAS, promotes cell growth at the expense of differentiation, and perturbs feedback regulation, leading to altered biochemical responses to the MEK inhibitor selumetinib.

Upstream inputs from RTKs to Ras such as the Ras GEFs SOS1 and SOS2 as well as the protein tyrosine phosphatase SHP2 encoded by the *PTPN11* gene have emerged as potential therapeutic targets for *NF1* mutant tumors (7–9, 11, 12). Indeed, multiple upstream regulators of Ras activation such as *SOS1, SOS2, PTPN11,* and *GAB1* exhibit significant negative correlations with *NF1* in the Cancer Dependency Map, supporting the importance of these genes as functionally opposing NF1 function (*SI Appendix*, Fig. S4*A* and Dataset S4). In iPNs, *SOS1, SOS2, PTPN11*, or the adapters *GAB1* and *GAB2* were all present and not differentially expressed in sg*NF1* cells (*SI Appendix*, Fig. S4*B*). In contrast, phosphoproteomic analysis revealed altered posttranslational regulation of SOS1, GAB2, and SHP2 in sg*NF1* iPNs: the inhibitory SOS1 serine 1167 (S1167) phosphorylation was significantly increased in sg*NF1* cells (*SI Appendix*, Fig. S4*C*) while the activating GAB2 serine 210 (S210) and SHP2 tyrosine 542 (Y542) phosphosites were significantly decreased in CRISPRi sg*NF1* iPNs (*SI Appendix*, Fig. S4 *D* and *E*). In sum, these observations suggest that *NF1* loss alters responses and decreases sensitivity to the MEK inhibitor selumetinib.

**Fig. 2.**
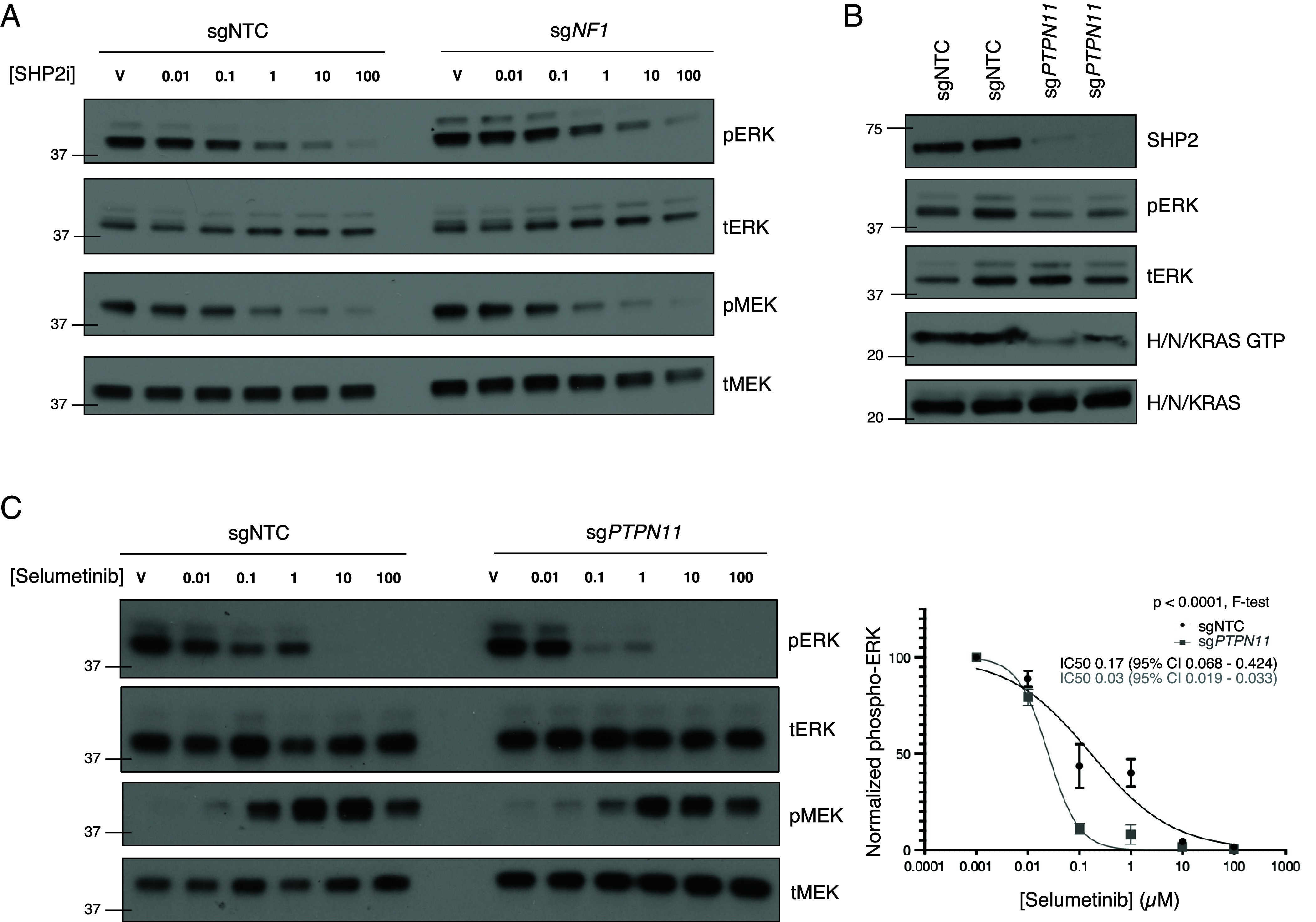
CRISPRi sg*PTPN11* repression alters cell proliferation/differentiation and increases sensitivity to the MEK inhibitor selumetinib. (*A*) There is no significant difference in SHP2 inhibitor dose–response in *sgNF1* compared to sgNTC control iPNs. (*B*) sg*PTPN11* iPNs show decreased pERK and Ras GTP levels. (*C*) sg*PTPN11* iPNs show significantly greater inhibition of pERK in response to the MEK inhibitor selumetinib compared to sgNTC iPNs.

We next focused on SHP2 inhibition, which is critical for upstream Ras GTP loading and evaluated the dose–response to the SHP2 inhibitor RMC 4550 (13, 14), finding CRISPRi sg*NF1* iPNs showed no difference in biochemical sensitivity compared to sgNTC iPNs (Fig. 2*A* and *SI Appendix*, Fig. S5*A*). To better understand the effects of SHP2 perturbation, we next generated stable CRISPRi sg*PTPN11-*deficient iPNs (*SI Appendix*, Fig. S5*B*), which exhibited significantly decreased cell growth compared to sgNTC iPNs (*SI Appendix*, Fig. S5*C*). Immunoblotting showed decreased phospho ERK activation and Ras GTP loading in sg*PTPN11* iPNs (Fig. 2*B*). To define the transcriptomic effects of sg*PTPN11* repression, we then performed RNA-sequencing of sg*PTPN11* iPNs (Dataset S1), identifying a total of 321 significantly repressed and 595 significantly induced genes (*SI Appendix*, Fig. S6*A*). Gene ontology analysis revealed genes induced in sg*PTPN11* iPNs were enriched for positive regulators of development and negative regulators of cell proliferation (*SI Appendix*, Fig. S6*B*), thus expressing the inverse molecular signatures compared to sg*NF1* iPNs. Finally, we tested the biochemical response to selumetinib in sgNTC compared to sg*PTPN11* iPNs. Immunoblotting showed that sg*PTPN11* cells were significantly more sensitive to selumetinib than control sgNTC iPNs (Fig. 2*C*). In sum, combined analysis of genetic *NF1* or *PTPN11* perturbation with MEK or SHP2 pharmacologic blockade suggests *NF1* loss on its own does not sensitize cells to SHP2 inhibition, but *PTPN11* repression instead leads to the opposite transcriptional programs induced by *NF1* repression with concomitant selumetinib sensitization.

**Fig. 3.**
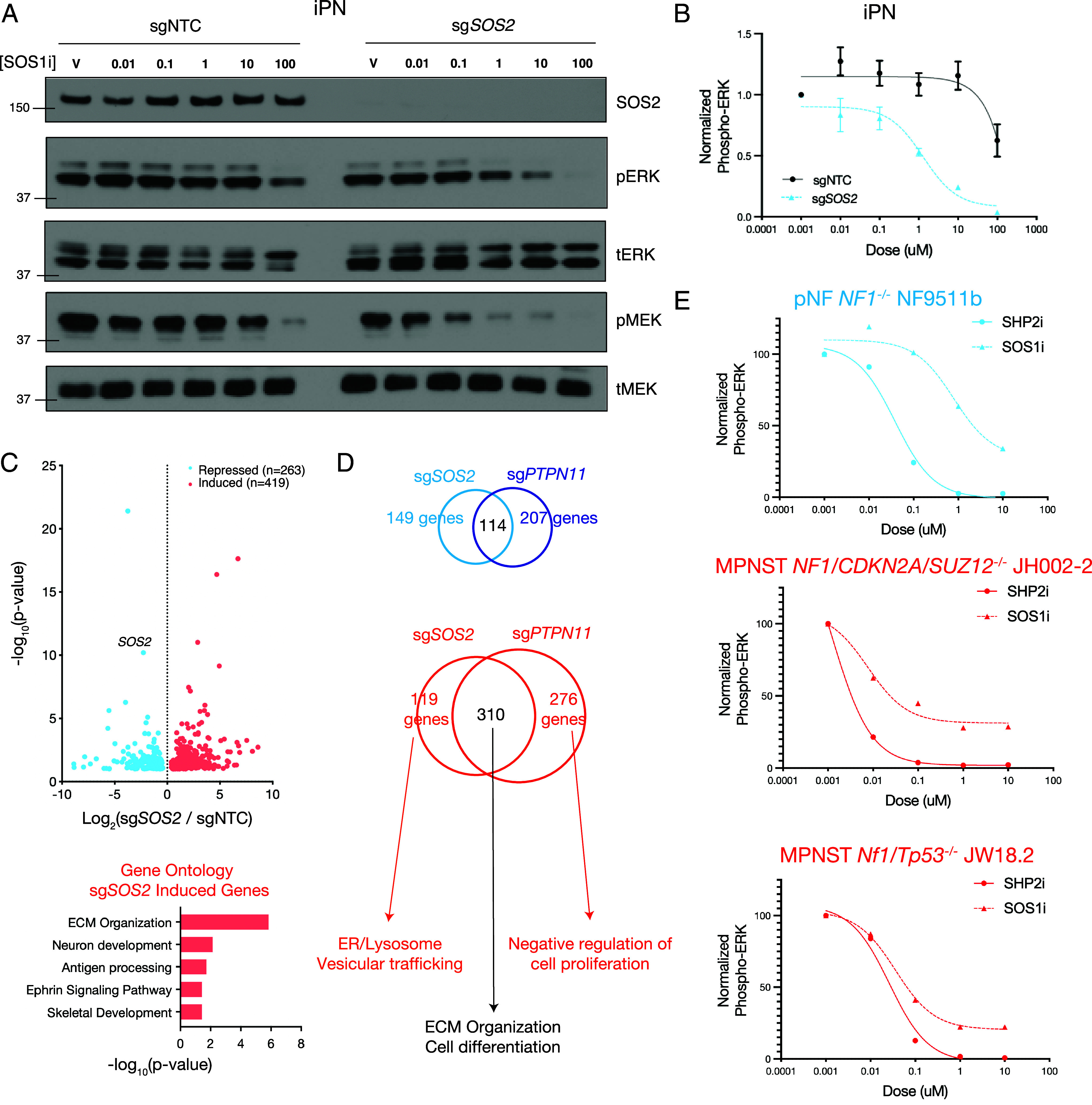
SOS1 inhibition is limited by SOS2 compensation and shows decreased biochemical response compared to SHP2 inhibition in *NF1* deficient cells. (*A* and *B*) sg*SOS2* iPNs demonstrate significantly improved response to SOS1 inhibition compared to sgNTC iPNs. (*C*) RNA-sequencing of sg*SOS2* iPNs identifies 263 significantly repressed genes (blue) and 419 significantly induced genes (red) with enrichment for ECM, differentiation, and Ephrin signaling gene sets. (*D*) Comparison of differential expression analysis between sg*PTPN11* and sg*SOS2* reveals shared and private gene expression programs highlighted by overlapping regulation of cell differentiation but a *PTPN11* specific effect on cell proliferation. (*E*) Biochemical analysis of pERK (*Top*) and pMEK (*Bottom*) responses to SHP2 inhibition (solid) or SOS1 inhibition (dashed) across a panel of pNF (blue) or MPNST (red) cells demonstrates greater efficacy of SHP2 inhibition compared to SOS1 inhibition.

Given the effects of genetic *PTPN11* and pharmacologic SHP2 perturbation, we next tested the biochemical effects of inhibiting the Ras GEF SOS1 (12). SOS1 inhibition showed limited biochemical efficacy in iPN cells (Fig. 3*A*). We hypothesized that this may be due in part to compensation by SOS2. We thus generated CRISPRi sg*SOS2* iPNs (*SI Appendix*, Fig. S7*A*) that showed no effect on cell growth (*SI Appendix*, Fig. S7*B*), in contrast to cell growth differences observed in sg*NF1* (*SI Appendix*, Fig. S1*B*) or sg*PTPN11* cells (*SI Appendix*, Fig. S5*C*). However, we observed a significantly improved response to SOS1 inhibition in sg*SOS2* iPNs compared to sgNTC control iPNs (Fig. 3 *A* and *B*). To define the transcriptomic consequences of *SOS2* repression, we performed RNA-sequencing of sg*SOS2* iPNs. We identified a total of 263 significantly repressed and 419 significantly induced genes, and gene ontology analysis revealed enrichment of cell differentiation programs enriched in genes induced upon sg*SOS2* repression (Fig. 3*C* and Dataset S1). To better understand the distinction between perturbation of upstream inputs via sg*PTPN11* or sg*SOS2* repression, we compared the gene set overlaps between sg*SOS2* and sg*PTPN11* cells (Dataset S5). Both sg*PTPN11* and sg*SOS2* deficient cells coordinately induced extracellular matrix (ECM) and cell differentiation gene programs. Sg*SOS2* cells alone affected genes associated with vesicular trafficking to the endoplasmic reticulum (ER) and lysosomal compartments, while sg*PTPN11* cells alone promoted cell proliferation transcriptional programs (Fig. 3*D*), consistent with the effect of sg*PTPN11* repression, but not sg*SOS2* repression, on iPN cell growth. We next tested the biochemical response to either SHP2 inhibition or SOS1 inhibition across a broader range of *NF1* mutant pNF (NF9511b) or MPNST (JH002-2, JW18.2) cell lines (*SI Appendix*, Fig. S8), revealing SHP2 inhibition showed a consistently superior biochemical response and underscoring the likely importance of SOS2 compensation in *NF1* mutant PNS tumor cells (Fig. 3*E*).

**Fig. 4.**
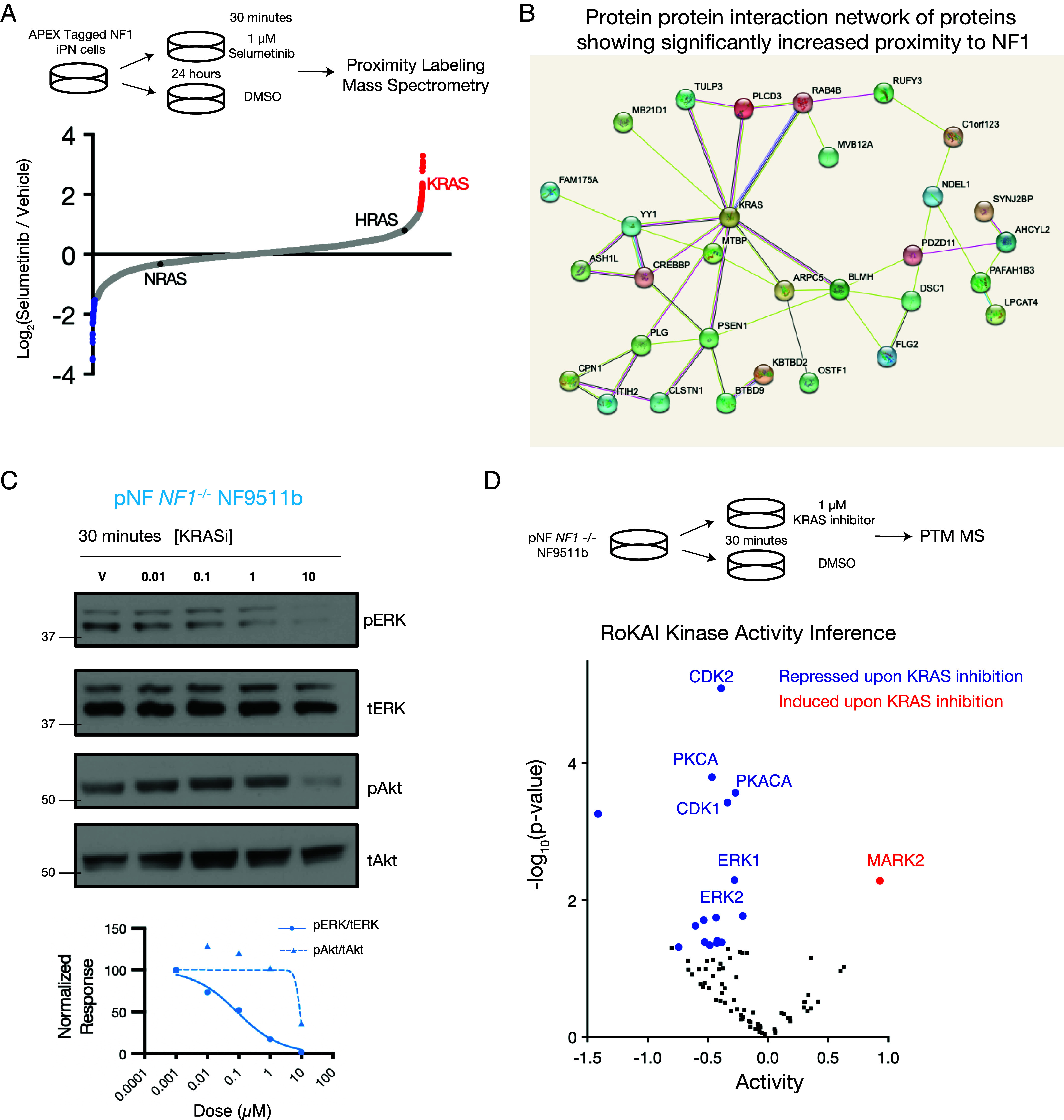
KRAS is a critical NF1 effector in the PNS. (*A*) APEX proximal proteomic mass spectrometry identifies KRAS, but not HRAS or NRAS, as significantly enriched in proximity to NF1 following selumetinib treatment (n = 44 significantly enriched proteins, red). (*B*) Protein–protein interaction network of 44 significantly enriched proximal proteins shows a KRAS-centric network. (*C*) The pan KRAS inhibitor preferentially blocks phospho-ERK in *NF1^−/−^* plexiform NF9511b neurofibroma cells with a modest effect on phosphoAkt only at 10 μM. (*D*) PTM-MS of *NF1^−/−^* plexiform NF9511b neurofibroma cells treated with 1 μM pan KRAS-inhibitor BI-2865 for 30 min reveals significantly decreased kinase activity of CDK1, CDK2, PKC, ERK1, and ERK2.

To identify neurofibromin interacting proteins in the PNS that may form the basis for additional pharmacologic approaches beyond MEK and SOS1/SHP2 inhibition, we next undertook a proximity labeling MS approach using APEX-tagged neurofibromin (15). The APEX tag did not affect NF1 localization in iPNs (*SI Appendix*, Fig. S9*A*), and evaluation across multiple doses of biotin phenol revealed multiple putative neurofibromin proximal proteins by immunoblot (*SI Appendix*, Fig. S9*B*). APEX proximity labeling MS for neurofibromin in PNS cells treated with 1 μM selumetinib compared to vehicle DMSO for 24 h revealed a total of 44 significantly increased proximal proteins and 43 significantly decreased proximal proteins following selumetinib treatment (*SI Appendix*, Fig. S10 and Dataset S6). Among Ras family small GTPases, only KRAS showed significantly increased proximity to neurofibromin (Fig. 4*A*), and protein–protein interaction network visualization further revealed KRAS was the most highly interconnected signaling node within significantly increased proteins (Fig. 4*B*). Consistent with this observation, analysis of CRISPRi screens for mediators of selumetinib sensitivity in *NF1* mutant pNF cells (6) showed that *KRAS* repression, but not *NRAS* or *HRAS* repression, resulted in significantly increased selumetinib sensitivity (*SI Appendix*, Fig. S11*A*), which could not be explained solely by expression of each Ras gene in iPN, pNF, or MPNST cells (6) (*SI Appendix*, Fig. S11*B*). Pan-KRAS inhibition (BI-2865) (16) demonstrated robust biochemical suppression of pERK in *NF1* mutant NF9511b pNF cells at 30 min, with an effect on pAKT observed only at higher doses (Fig. 4*C*). Finally, PTM-MS following treatment of *NF1* mutant NF9511b pNF cells with 1 μM pan KRAS inhibitor (BI-2865) identified 49 significantly downregulated phosphopeptides and 144 significantly upregulated phosphopeptides (*SI Appendix*, Fig. S12*A* and Dataset S7). Kinase enrichment analysis revealed significant suppression of CDK1/2 and ERK1/2, suggesting KRAS inhibition is sufficient to block RAF/MEK/ERK activation and cell cycle progression in *NF1* mutant pNF cells (Fig. 4*D* and *SI Appendix*, Fig. S12*B*). Interestingly, PKC activity was also robustly suppressed by KRAS inhibition, potentially underscoring a role for blocking KRAS activation indirectly through PKC inhibition. Taken together, these data suggest the key catalytic target of neurofibromin GAP activity in the PNS is KRAS, which can be therapeutically leveraged using pan KRAS inhibitors.

## Discussion

MEK inhibitors are approved for NF-1-associated tumors, yet the therapeutic window, highlighted by partial responses and reported toxicity, remains a challenge (4, 17, 18). Here, we combined CRISPRi PNS cell models with proteomic, transcriptomic, and pharmacologic approaches to define the response to perturbation of multiple upstream and downstream Ras signaling nodes in relation to neurofibromin. Our phosphoproteomic analysis reveals a dampened response to MEK inhibition in CRISPRi sg*NF1* iPNs, suggesting the therapeutic window in people with NF-1, in which *NF^−/−^*tumor cells exist within a heterozygous *NF1^+/−^* microenvironment, requires careful consideration to optimize the differential response between *NF1* intact and *NF1-*deficient cells. Upstream perturbation of SHP2 encoded by the *PTPN11* gene showed a greater effect than SOS1 inhibition, the latter of which appears to be due to compensation by SOS2. Consistent with this observation, CRISPRi sg*PTPN11* iPNs display the inverse transcriptomic signature to sg*NF1* iPNs, which only partially overlaps with the transcriptomic changes observed in sg*SOS2* iPNs. Finally, neurofibromin APEX proximity labeling mass spectrometry identifies KRAS as the key Ras family small GTPase regulated by neurofibromin in iPNs consistent with functional genomic approaches to identify mediators of selumetinib response. Genetic or pharmacologic suppression of KRAS blocks RAF/MEK/ERK in *NF1* mutant pNF cells. Taken together, our data identify multiple alternate therapeutic approaches for *NF1* mutant tumors beyond MEK inhibition, underscoring both potential benefits and limitations of upstream blockade and ultimately converging on KRAS as the critical therapeutic target in the *NF1* mutant PNS.

Growth factor signaling through RTKs and Ras is tightly regulated by multiple waves of feedback (19, 20). The MEK inhibitors selumetinib (4, 17) and mirdametinib (5) demonstrate clinical efficacy for *NF1* mutant pNFs, yet resistance to MEK inhibition often arises through feedback reactivation of RAF/MEK/ERK signaling (21). Indeed, our data suggest that the *NF1* intact microenvironment cells may be more sensitive to MEK inhibition than *NF1^−/−^* tumor cells at least in part due to how *NF1* loss modulates Ras pathway activation and feedback regulation. One proposed strategy to overcome this limitation is through serial upstream inhibition of Ras activation by blocking Ras GTP loading by SOS GEFs (9, 22, 23) which requires SHP2 (13, 24, 25). Indeed, such strategies testing combination SOS1 (9) or SHP2 inhibition (7, 8) have shown promise in multiple preclinical studies for *NF1* mutant pNFs and MPNSTs. Our work complements these efforts through genetic perturbations and transcriptomic profiling that reveal sg*PTPN11* repression leads to the opposite gene expression signature to sg*NF1* repression, suggesting that neurofibromin’s GAP activity is opposed by SHP2-dependent Ras GEF activity In conjunction with the Cancer DepMap anticorrelations between *NF1* and *PTPN11* (and to a lesser degree *SOS1* and *SOS2*), these data strongly reinforce the model that neurofibromin’s dominant catalytic function is to serve as a Ras GAP, with additional neurofibromin interactors serving primarily to regulate its GAP activity. One such example is its interaction with SPRED proteins, which are observed to be neurofibromin’s top correlated genes in the Cancer DepMap. We further find that while *NF1* repression does not preferentially sensitize iPNs to SHP2 inhibition, sg*PTPN11* iPNs are more sensitive to MEK inhibition, and SHP2 inhibition generally is more effective than SOS1 inhibition across a panel of MPNST cells likely due to compensation by SOS2.

The advent of Ras inhibitors (26) has opened the door to leveraging this class of therapeutic agents for all Ras-driven tumors, including those harboring *NF1* loss without an oncogenic Ras variant. From a biochemical perspective, neurofibromin’s role as a Ras GAP for the three classical Ras proteins (KRAS, HRAS, and NRAS) is well established (3, 27), yet the precise Ras protein(s) catalytically regulated by neurofibromin may vary or be redundant across different cell types and disease contexts, leaving the context specific clients of neurofibromin incompletely understood. Consistent with this notion of context specificity and potential promiscuity, prior work has suggested neurofibromin may regulate different, or multiple, Ras proteins across different cell lineages: Neurofibromin primarily acts on both HRAS and KRAS in melanoma (28) while KRAS alone is the critical effector in mast cells (29) or glioma (30). Our work provides proteomic, functional genetic, and pharmacologic evidence that KRAS is the key PNS effector of neurofibromin. This does not appear to be solely due to gene expression, as all three classical Ras genes (*H/N/KRAS*) are expressed in iPN, pNF, and MPNST cells. The mechanism of Ras GTPase specificity for neurofibromin, and by extension, other Ras GAPs, will be an important area for future investigation and may be due to posttranscriptional regulation, differences in GTP loading, subcellular localization, and/or differences in contacts between neurofibromin and each Ras protein outside the conserved Ras–GRD interface. Finally, the observation that MEK inhibition promotes the association between neurofibromin and KRAS in our APEX experiment suggests that Ras/RAF/MEK inhibition may promote a positive feedback loop to drive NF1 mediated hydrolysis of active Ras-GTP, and identifying the precise proteins and interactions underlying this feedback loop may lead to additional insights regarding the regulation of neurofibromin activity. In sum, these observations support the use of KRAS inhibition, potentially obviating the need for pan Ras inhibition (31) and thus potentially providing an alternate targeted therapeutic approach for people with NF1 associated pNFs. Building on the clinical success of MEK inhibition, which inhibits downstream outputs from KRAS, HRAS, and NRAS, directly targeting KRAS may provide a cleaner therapeutic window given it is the direct catalytic target of neurofibromin, offering an alternate treatment for people who do not respond to or cannot tolerate MEK inhibitors.

Our study has a number of limitations, many of which are inherent to current PNS cell culture models such as iPNs, the NF9511b pNF cells, and the set of MPNST cells used in this study that may not fully recapitulate the genetic architecture or biochemical dependencies observed in vivo. Moreover, our data do not preclude partial compensation or functional redundancy by other Ras proteins, particularly following KRAS blockade, although it is notable neither *NRAS* or *HRAS* emerged from our genome wide CRISPRi screen to identify mediators of selumetinib response in *NF1* mutant pNF cells (6). Similarly, our data leave open the question of how resistance to KRAS inhibition may arise and whether a pan Ras inhibitor may circumvent feedback reactivation through other Ras proteins. Nevertheless, it is clear that direct Ras inhibition in some capacity will likely prove beneficial in *NF1* mutant tumors and other cancers driven by inappropriate activation of wildtype Ras proteins.

## Materials and Methods

### CRISPRi Cell Line Generation.

Lentivirus containing pMH0001 (UCOE-SFFV-dCas9-BFP-KRAB, #85969, Addgene) was produced from transfected HEK293T cells with packaging vectors (pMD2.G #12259, Addgene, and pCMV-dR8.91, Trono Lab) following the manufacturer’s protocol (#MIR6605, Mirus) as previously reported (6). IPN cells were stably transduced to generate parental iPN^dCas9-KRAB-BFP^ cells and selected by flow cytometry using a SH800 sorter (Sony). Subsequent gene-specific knockdowns were achieved by individually cloning single-guide RNA protospacer sequences into the pCRISPRia-v2 vector (#84832, Addgene) between BstXI and BlpI restriction sites. All constructs were validated by Sanger sequencing of the protospacer region. The following protospacers were used: sgNTC (GTGCACCCGGCTAGGACCGG), sg*NF1*-1 (GGGATCCTTTCCACGGCCCG), sg*NF1*-2 (GGTGGGGAGACGGCGCTAGT), sg*PTPN11*-1 (GGAGGAACATGACATCGCGG), sg*PTPN11*-2 (GGCAAGGAGCGGGTCCGTCG), sg*SOS2*-1 (GCCAGCGCCGCCGGGAAAGG), sg*SOS2*-2 (GCACAGGGCTCGAGTCCCCG). Lentivirus was generated as described above, and cells were selected to purity using 1 μg/mL puromycin for at least 5 d.

### Cell Culture, Cell Viability Assays, and In Vitro Pharmacology.

Patient-derived iPN, neurofibroma (NF95.11b), or human MPNST (JH002-2, ST88-14) cell lines were obtained from the Neurofibromatosis Therapeutic Acceleration Program or American Type Culture Collection. Mouse MPNST cell lines (JW18.2, JW23.3) were a gift from Angie Hirbe (Washington University in St. Louis, MO). Cell lines were grown in Dulbecco’s Modified Eagle Medium (#11960069, Life Technologies) with 10% FBS and 1× Pen-Strep (#15140122, Life Technologies) as previously reported (6). Cell lines were regularly tested and verified to be *Mycoplasma* negative (#LT07-218, Lonza). Viability assays were carried out with the CellTiter 96 Non-Radioactive Cell Proliferation Assay (#G410, Promega) and a Glomax Discovery Multimode Microplate Reader (Promega). For pharmacologic assays, cells were seeded at a density of 5,000 cells per well in a 96-well plate the night prior to treatment, after which cells were treated with drugs at the indicated concentrations for the indicated periods (or 48 h if not otherwise indicated) prior to experimentation.

### Nucleic Acid Extraction for Bulk RNA Sequencing.

RNA were isolated from cell using the All-Prep Universal Kit (#80224, QIAGEN). QiaCubes were used for standardized automated nucleic acid extraction as per the manufacturer’s protocol as previously reported (6). For cell line samples, pellets were directly lysed in RLT Plus buffer with beta-mercaptoethanol. RNA quality was assessed by chip-based electrophoresis on a BioAnalyzer 2100 using the RNA 6000 Nano Kit (#5067-1511, Agilent Technologies), and clean-up was performed as needed using the RNeasy kit (QIAGEN). Only samples with high-quality and/or RNA (RIN > 8) were used for bulk RNA sequencing.

### RNA Sequencing.

Library preparation was performed using the TruSeq RNA Library Prep Kit v2 (#RS-122- 2001, Illumina), and 50 bp single end reads were sequenced on an Illumina HiSeq 2500 or NovaSeq to a minimum depth of 25 M reads per sample at Medgenome, Inc. Quality control of FASTQ files was performed with FASTQC, and after trimming of adapter sequences, reads were filtered to remove bases that did not have an average quality score of 20 within a sliding window across four bases (http://www.bioinformatics.babraham.ac.uk/projects/fastqc/) as previously reported (6). Reads were mapped to the appropriate reference genome (hg19) using HISAT2 with default parameters (32). Transcript abundance estimation in transcripts per million (TPM) and differential expression analysis were performed using DESeq2 (33). Differentially expressed transcripts with an adjusted *P*-value < 0.1 were identified and filtered based on an expression cutoff (TPM > 1) and a fold change threshold (log_2_FC > 1) to prioritize biologically relevant gene sets. Clustering dendrograms and heatmaps were generated in R using TPM values and plotted as normalized row expression values with the heatmaps.2 function.

### Immunoblotting.

Whole-cell lysates were harvested using radioimmunoprecipitation assay (RIPA) buffer (50 mM Tris-HCl at pH 8.0, 150 mM NaCl, 0.5% Deoxycholate, 0.1% Sodium Dodecyl Sulfate (SDS), 1% IGEPAL CA-630) with fresh protease (#P8340, Sigma) and phosphatase inhibitor (#P2850, Sigma) cocktails as previously reported (6). To pull down GTP-bound RAS, supernatants from cleared lysates were incubated with recombinant GST-RAF1 RBD beads for 2 h at 4 °C followed by three washes with lysis buffer but without protease or phosphatase inhibitors. Beads and lysates were mixed with Nu polyacrylamide gel electrophoresis (NuPAGE) LDS (ThermoFisher) sample buffer and heated at 70 °C for 10 min. A total of 10 to 20 μg of protein was loaded into precast NuPAGE electrophoresis gels (Life Technologies). Samples were separated by SDS-PAGE, transferred to nitrocellulose or polyvinylidene fluoride membranes, and blocked in either 5% bovine serum albumin or 5% skim milk in TBS buffer for 1 h at room temperature. Primary antibodies were incubated overnight at the indicated dilutions at 4 °C and horseradish peroxidase conjugated secondary antibodies were incubated for 1 h at room temperature followed by ECL based detection on film. The following antibodies were used: pERK (Cell Signaling Technologies, #4370, 1:1,000 dilution), total ERK (Cell Signaling Technologies, #4695, 1:1,000 dilution), pAKT (Cell Signaling Technologies, #4060, 1:1,000 dilution), total AKT (Cell Signaling Technologies, # 4685, 1:1,000 dilution), pMEK (Cell Signaling Technologies, #9121, 1:1,000 dilution), total MEK (Cell Signaling Technologies, #8727, 1:1,000 dilution), neurofibromin (Millipore, #3668175, 1:1000 dilution), SHP2 (Cell Signaling Technologies, #3397, 1:1,000 dilution), and SOS2 (Santa Cruz, sc-393667). Quantification was performed in ImageJ (NIH) using the relative densitometry between phosphorylated and total protein abundance from immunoblotting.

### qRTPCR.

RNA was extracted from cell lines using the RNeasy Mini Kit (#74106, QIAGEN) according to the manufacturer’s instructions, and complementary DNA (cDNA) was synthesized from RNA using iScript cDNA Synthesis kit (#1708891, Bio-Rad). Real-time QPCR was performed using PowerUp SYBR Green Master Mix (#A25918, Thermo Fisher Scientific) on a QuantStudio 6 Flex Real Time PCR system (Life Technologies). The following QPCR primers were used: GAPDH-F (5′-GTCTCCTCTGACTTCAACAGCG-3′), GAPDH-R (5′-ACCACCCTGTTGCTGTAGCCAA-3′), NF1-F (5′-CGAATCATCACCAATTCCGCA-3′), NF1-R (5′-CCACAACCTTGCACTGCTTTAT-3′), PTPN11-F (5′-GAACTGTGCAGATCCTACCTCT-3′), PTPN11-R (5′-TCTGGCTCTCTCGTAC AAGAAA-3′), SOS2-F (5′- CCGCAGCCTTACGAGTTCTTC-3′), or SOS2-R (5′-GGATGCAC TTGTTCCTGAACC-3′).

### Posttranslational Modification Mass Spectrometry.

Cells were washed 3× in ice-cold phosphate buffered saline, scraped and pelleted, and snap-frozen in dry ice until processing for mass spectrometry. To prepare peptides for phosphoenrichment, cell pellets were lysed in 8 M urea + 25 mM ammonium bicarbonate followed by reduction (5 mM dithiothreitol (DTT) for 1 h at 37 °C), alkylation (10 mM iodoacetamide for 30 min at room temperature in the dark), and digestion overnight with mass spectrometry grade Trypsin (Promega, #V5111) and Lysyl Endopeptidase (Fujifilm Wako, #125-02543) at a ratio of 100:1 protein to enzyme. Digested peptides were applied to activated C18 columns, and columns were washed three times with 1mL of 0.1% trifluoroacetic acid (TFA). Peptides were eluted with 600 µL of 50% Acetonitrile (ACN) and 0.25% formic acid (FA). Peptides were quantified using Pierce™ Quantitative Peptide Assay (ThermoFisher Scientific, #23275) and peptide amounts were normalized prior to lyophilization. Phosphoenrichment was performed on peptides using PTMScan® Phospho-Enrichment IMAC Fe-NTA Magnetic Beads (Cell Signaling Technology, #20432) as per the manufacturer’s protocol.

Samples were resuspended in 0.1% FA and separated by reversed-phase chromatography using a Vanquish Neo high-performance liquid chromatography (HPLC) instrument (Thermo Fisher Scientific) with a 15-cm PepSep column (150 µm inner diameter) packed with 1.5-µm Reprosil Saphir C18 particles (Bruker). Samples were acquired by data-dependent acquisition (DDA). Mobile phase A consisted of 0.1% FA in water and mobile phase B consisted of 80% ACN and 0.1% FA. Peptides were separated at a flow rate of 600 nL min^−1^ over the following 80-min gradient: 3 to 28% B in 67 min, 28 to 40% B in 5 min, and 8 min at 95% B. Eluting peptides were analyzed by an Orbitrap Exploris MS instrument (ThermoFisher Scientific). Data were collected in positive ion mode with MS1 resolution of 120,000, 350 to 1,250 m/z scan range, auto maximum injection, and radiofrequency lens of 50%. For DDA, MS2 fragmentation was performed on charge states 2 to 6 with a 20-s dynamic exclusion after a single selection and 10 ppm ± mass tolerance. Raw mass spectrometry data were searched using Fragpipe (v22) (34, 35) against the human proteome (UniProt canonical protein sequences, downloaded in September 2022) using PhosphoLFQ (36–38) label-free quantification workflow. Statistical analysis was performed using MSstats (v4.8.7) (39) from the artMS (v1.18.0) R package. Kinase enrichment analysis was performed using RoKAI Differential Expression analysis (40).

For samples run by data independent acquisition (DIA), samples were resuspended in 0.1% formic acid and separated by reversed-phase (RP) chromatography using a Thermo EASY-nLC instrument (Thermo Fisher Scientific) with a 15-cm PepSep column (150 µm inner diameter) packed with 1.5-µm Reprosil Saphir C18 particles (Bruker). Mobile phase A consisted of 0.1% FA in water and mobile phase B consisted of 80% ACN and 0.1% FA. Peptides were separated at a flow rate of 1.5ul min−1 over the following 110-min gradient: 2–15% B in 88 min, 15–25% B in 10 min and 12 min at 90% B. Eluting peptides were analyzed by an Orbitrap Lumos MS instrument (ThermoFisher Scientific). An MS scan was performed at 120,000 resolving power over a scan range of 300-1200 m/z, a standard AGC target of 100%, an RF lens setting of 30%, and an auto maximum injection time was acquired, followed by DIA scans using 16 m/z isolation windows over 358-1111 m/z at normalized HCD collision energies of 27, 32, and 37%. Loop control was set to All. A library was generated using Data Dependent Analysis (DDA). Samples were resuspended in 0.1% formic acid and separated by reversed-phase (RP) chromatography using a Thermo EASY-nLC instrument (Thermo Fisher Scientific) with a 15-cm PepSep column (150 µm inner diameter) packed with 1.5-µm Reprosil Saphir C18 particles (Bruker). Mobile phase A consisted of 0.1% FA in water and mobile phase B consisted of 80% ACN and 0.1% FA. Peptides were separated at a flow rate of 600nl min−1 over the following 95-min gradient: 3–30% B in 75 min, 30–38% B in 10 min and 10 min at 8% B. Eluting peptides were analyzed by an Orbitrap Lumos MS instrument (ThermoFisher Scientific). DDA was performed by acquiring a full scan over an m/z range of 375-1150 in the Orbitrap at 60,000 resolving power (@200 m/z) with a standard AGC target of 100%, an RF lens setting of 30%, and an auto maximum ion injection time. Dynamic exclusion was set to 60 seconds, with a 10 ppm exclusion width setting. Peptides with charge states 2-7 were selected for MS/MS interrogation using isolation width of 1.4m/z, and stepped HCD collision energy of 15, 30, 45%.

### APEX Reaction and Capture of Biotinylated Proteins.

Cells were incubated with 500 μM biotin-phenol in complete medium for 30 min at 37 °C. APEX labeling was initiated by addition of H_2_O_2_ at a final concentration of 1 mM and incubated for 30 s. To quench the reaction, H_2_O_2_ solution was discarded, and cells were immediately washed 3× with ice-cold quenching solution (10 μM Sodium ascorbate, 5 mM Trolox, 10 μM NaN_3_) for 1 min × wash. Cells were scraped and collected in quencher solution and pelleted. Excess solution was removed and cell pellets were snap-frozen. To proceed with pulldown of biotin-labeled proteins, cell pellets were lysed in RIPA buffer (150 mM NaCl, 50 mM Tris-HCl pH 7.5, 0.5% sodium deoxycholate, 0.1% SDS, 1% Triton-X100) supplemented with 10 μM Sodium ascorbate, 5 mM Trolox, 10 μM NaN_3_, 1 mM DTT, and protease inhibitor (Complete protease inhibitor, Sigma #4693159001). Cell lysates were sonicated 3× at 15% for 7 s. Lysates were cleared at 13,000 g for 10 min at 4 °C and protein concentrations were quantified using 600 nM Pierce Assay Kit with Detergent Compatibility Reagent (ThermoFisher, #22662). Streptavidin magnetic beads (ThermoFisher, #88816) were washed 2× with RIPA buffer and 100 μL washed beads were added per sample. Binding was allowed to proceed overnight at 4 °C with rocking. Beads were collected on a magnetic rack and the following wash steps were performed at 4 °C: 3× wash with RIPA buffer (5 min each), 1× wash with 1 M KCL (2min), 1× wash with Na_2_CO_2_ (2min), and 2× with 50 mM Tris-HCl [pH = 8.0] (5 min). Beads were resuspended in 200 μL Digestion buffer (2 M urea in 50 mM Tris pH = 8.0). Samples were reduced by adding 5 mM Tris(2-carboxyethyl)phosphine and incubated at 37 °C for 30 min with orbital shaking at 1,000 rpm. Samples were alkylated with iodoacetamide at 5 mM at room temperature, covered from light with orbital shaking at 1,000 rpm. The reaction was quenched with addition of 5 mM DTT. Biotinylated proteins were digested with addition of 0.8 μg mass spectrometry grade Trypsin (Promega, #V5111) overnight at 37 °C with orbital shaking at 1,000 rpm. The following morning, an additional 0.4 μg trypsin was added and incubated for 2 h at 37 °C with shaking. Samples were acidified with 0.5% TFA and desalted using C18 96-well plate format (Higgins Analytical, #96WR-20mg-W182).

### APEX Proximity Labeling Mass Spectrometry.

APEX-labeled samples were acquired on a Bruker TimsTOFpro interfaced with an Ultimate3000 UHPLC. Peptides were resuspended in 0.1% FA and were separated using a 60 min linear gradient. Following loading, the percentage of B (80% ACN in 0.1% FA) was increased from 2 to 26% in 49 min and then to 90% remaining time. The peptides were separated on a PepSep column (15 cm, 150 mm iid, 1.9 μm beads size). The mass spectrometer was operated in positive mode using DIA-PASEF acquisition (41). The mass range was set at 100 to 1,700m/z with 10 scans between 0.7 Vs/cm2 and 1.4 Vs/cm2. 2 ms and 100 ms were set for the accumulation and ramp times, respectively. Raw mass spectrometry files were analyzed using Spectronaut (version 18.4.231017.55695) against the human proteome (UniProt canonical protein sequences downloaded in September 2022) using default settings for spectral library generation and DIA. Statistical analysis was performed using MSstats (v4.8.7) (39) from the artMS (v1.18.0) R package. Visualization was performed in Cytoscape with additional connections included from the STRING database (42).

### Statistical Analysis.

All experiments were repeated as independent biologic replicates. Statistics were derived from biologic replicates, and no statistical methods were used to predetermine sample size. Cells were randomized to experimental conditions, and no data points were excluded from analysis. Unless otherwise specified, data are plotted as mean with error bars representing the SEM. The statistical tests of choice were selected based on the input data. All statistical tests were one-sided. Where appropriate, multiple hypothesis testing corrections were performed. Statistical significance thresholds are indicated in each figure legend and exact *P*-values are provided when possible.

## Supplementary Material

Appendix 01 (PDF)

Dataset S01 (XLSX)

Dataset S02 (XLSX)

Dataset S03 (XLSX)

Dataset S04 (XLSX)

Dataset S05 (XLSX)

Dataset S06 (XLSX)

Dataset S07 (XLSX)

## Data Availability

The mass spectrometry proteomics data have been deposited to the ProteomeXchange Consortium (http://proteomecentral.proteomexchange.org) via the PRIDE partner repository with the dataset identifiers: PXD064557 (43), PXD064434 (44), and PXD061467 (45).
