# Supplementary material for: A transcriptomic, proteomic, and functional genetic atlas dissects neurofibromin function in the peripheral nervous system
Source: Proc Natl Acad Sci U S A. 2025 Jun 30;122(27):e2506823122. doi: 10.1073/pnas.2506823122 (PMC12260521; doi:10.1073/pnas.2506823122)
Supplement: Supplementary file 1 — Appendix 01 (PDF) [file pnas.2506823122.sapp.pdf]

PNAS

Supporting Information for

A transcriptomic, proteomic, and functional genetic atlas dissects neurofibromin function in the peripheral nervous system

Harish N. Vasudevan,[1,2#] Nadia Arang,[3,4] Maria Sacconi Nunez,[1,2] Patrick Kennedy,[1,2] Emily Payne,[1,2] Sarah Mohabeer,[1,2,5] Julian Chien,[1,2] Aaron Wright,[1,2] Matthew J. Sale,[5] Nevan J. Krogan,[3,4] Antoine Forget,[3,4] Frank McCormick[5#]

[1]Department of Radiation Oncology, University of California San Francisco, San Francisco, CA 94143, USA.
[2]Department of Neurological Surgery, University of California San Francisco, San Francisco, CA 94143, USA.
[3]Quantitative Biosciences Institute (QBI), University of California, San Francisco, CA, USA.
[4]Department of Cellular and Molecular Pharmacology, University of California San Francisco, San Francisco, CA, USA.
[5]Helen Diller Family Comprehensive Cancer Center, University of California San Francisco, San Francisco, CA, USA.

# Corresponding authors
#Harish Vasudevan
Email: harish.vasudevan@ucsf.edu

#Frank McCormick
Email: frank.mccormick@ucsf.edu

This PDF file includes:

Figures S1 to S12
Legends for Datasets S1 to S7

Other supporting materials for this manuscript include the following:

Datasets S1 to S7

Supplementary Figures.

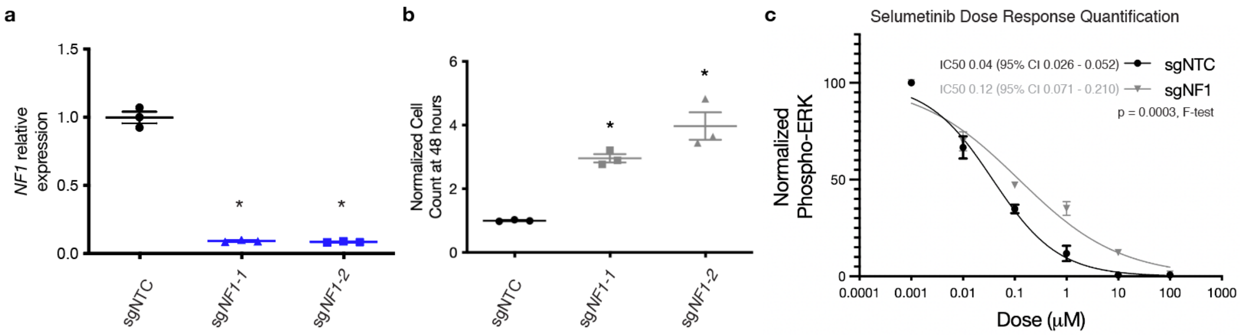

Figure S1. CRISPRi sg*NF1* deficient iPNs exhibit increased cell growth and decreased selumetinib response. a. Quantitative reverse transcription PCR (qRT-PCR) confirms repression of *NF1* in CRISPRi sg*NF1* iPNs. * $p < 0.05$, Student's t-test. b. CRISPRi sg*NF1* iPNs exhibit significantly increased growth compared to control CRISPRi sgNTC iPNs. * $p < 0.05$, Student's t-test. c. CRISPRi sg*NF1* iPNs show decreased pERK repression in response to selumetinib compared to control CRISPRi sgNTC iPNs.

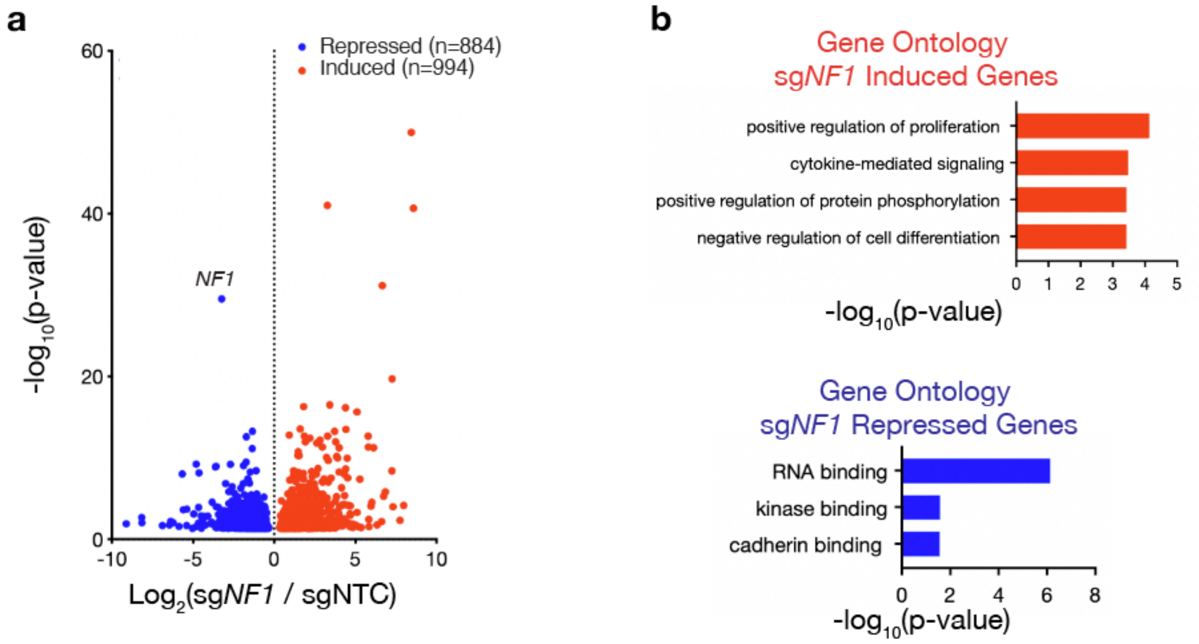

Figure S2. RNA-sequencing analysis reveals CRISPRi sg*NF1* repression is sufficient to promote proliferation, inhibit differentiation, and block expression of genes involved in feedback signaling. a. RNA-sequencing of sg*NF1* iPNs compared to sgNTC iPNs reveals 884 significantly repressed (red) and 994 significantly induced (blue) genes. b. Gene ontology analysis of induced genes reveals enrichment for cell proliferation, negative feedback regulators, and de-differentiation genes while repressed genes are enriched for regulators of RNA binding and kinase signaling genes.

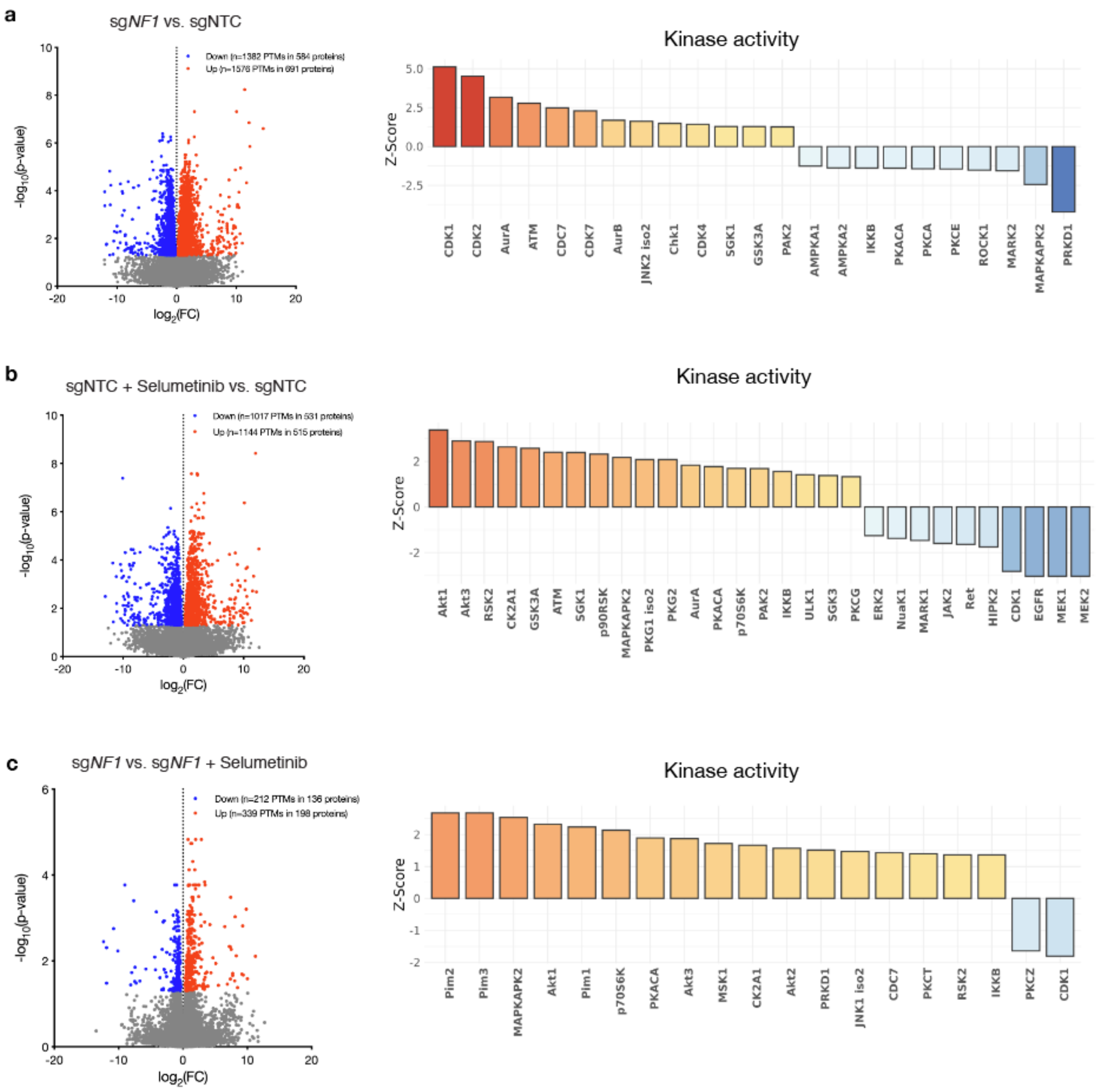

Figures S3. PTM-MS pairwise comparisons of sg*NF1,* sgNTC + selumetinib, or sg*NF1* + selumetinib compared to sgNTC iPNs reveal distinct phosphoproteomic alterations. a. sg*NF1* loss leads to induction of cyclin dependent kinases (CDKs) consistent with increased cell growth. b. Selumetinib treatment in sgNTC iPNs robustly represses MEK1/2, CDK1, and ERK activity with concomitant induction of AKT. c. Selumetinib treatment of sg*NF1* iPNs no longer repressed pMEK with a more modest effect on CDK1.

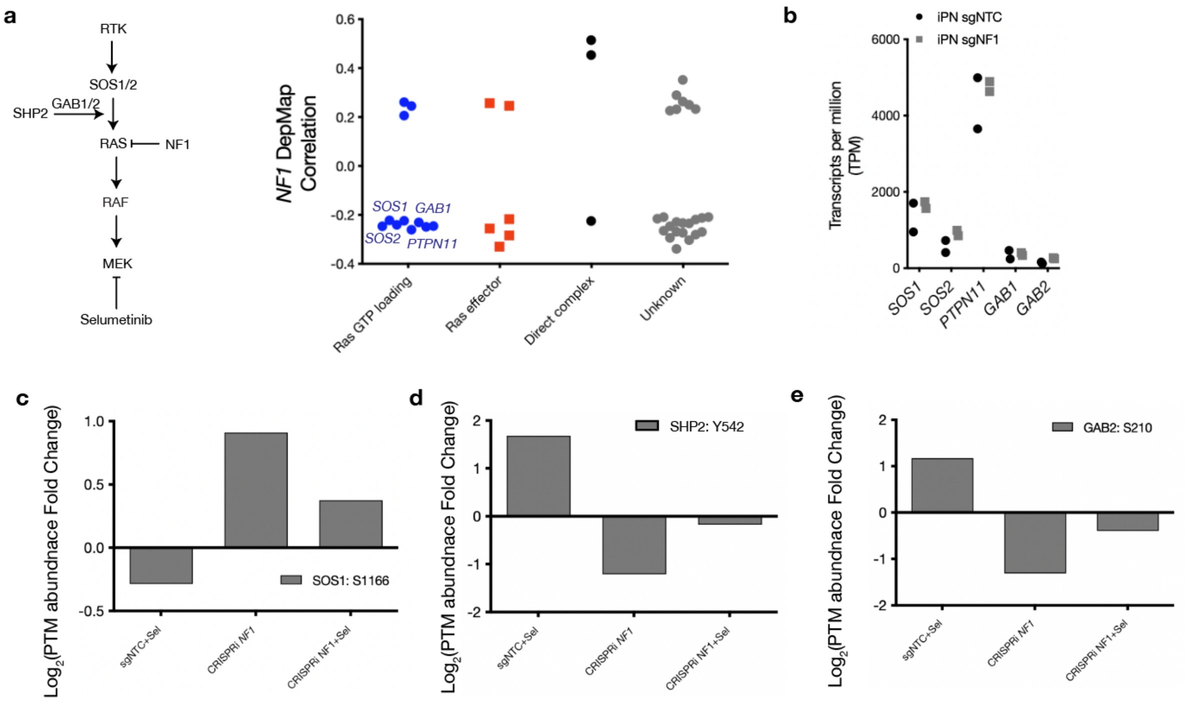

Figure S4. CRISPRi sg*NF1* iPNs display altered regulation of upstream inputs linking receptor tyrosine kinase to Ras signaling in response to selumetinib. a. Simplified diagram of receptor tyrosine kinase (RTK) mediated Ras activation through the Ras GEFs SOS1 and SOS2 potentiated by the tyrosine phosphatase SHP2 and the adapters GAB1 and GAB2. Cancer Dependency Map (https://depmap.org/) analysis of genes significantly correlated with *NF1* reveals multiple upstream inputs regulating Ras activation. b. *SOS1, SOS2, PTPN11, GAB1,* and *GAB2* are express in iPNs and not differentially expressed at the mRNA level following sg*NF1* repression. c. The repressive S1166 SOS1 phosphosite is increased in sg*NF1* compared to sgNTC iPNs and repressed upon selumetinib in both sgNTC and sg*NF1* iPNs, albeit to a lesser magnitude in sg*NF1* cells. d. The activating S210 GAB2 and e. Y542 SHP2 phosphosites are decreased in sg*NF1* compared to control cells and induced upon selumetinib in both sgNTC and sg*NF1* iPNs, albeit to a lesser magnitude in sg*NF1* cells.

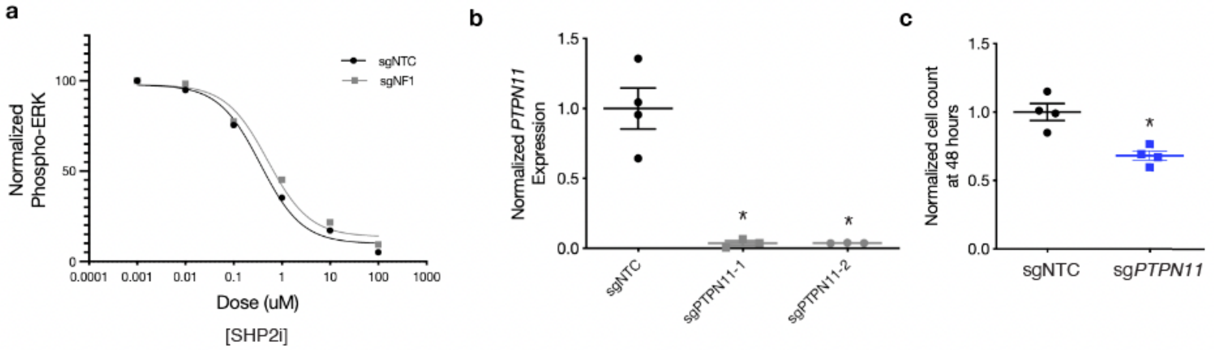

Figure S5. CRISPRi sg*NF1* cells show no difference in SHP2 inhibitor response, and *sgPTPN11* repression significantly decreases cell growth in iPNs. a. There is no significant difference in phospho ERK inhibition following SHP2 inhibitor treatment in sg*NF1* compared to sgNTC iPNs. b. qRT-PCR validation of sg*PTPN11* iPN cells. c. CRISPRi sg*PTPN11* repression is sufficient to significantly decrease cell growth. * $p < 0.05$, Student's t-test.

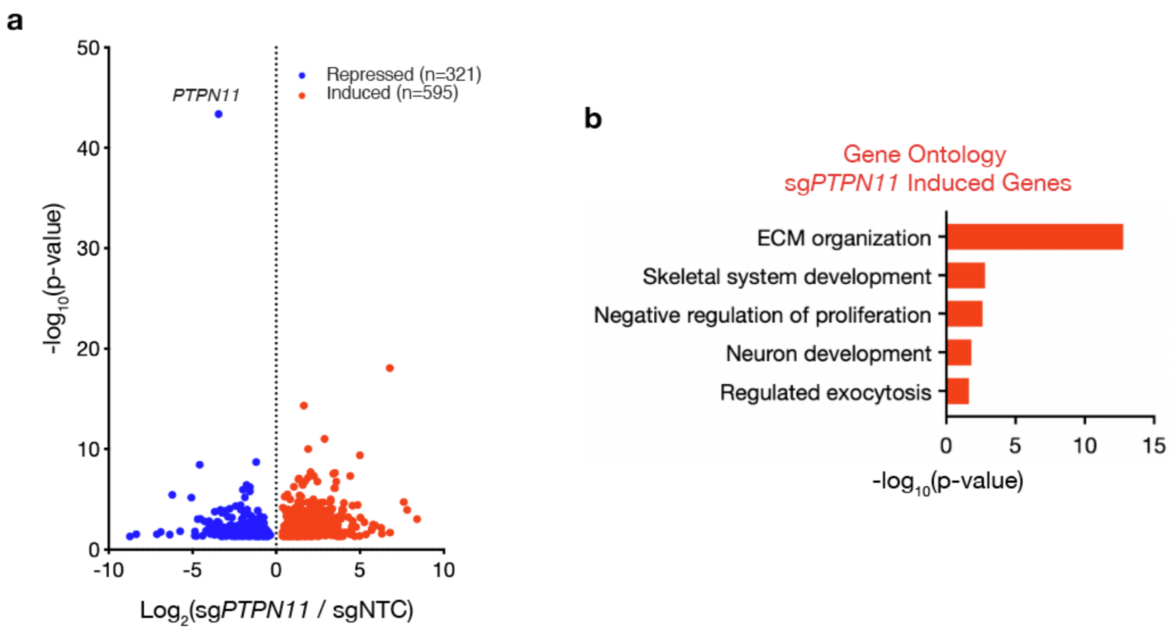

Figure S6. RNA-sequencing analysis reveals CRISPRi sg*PTPN11* repression is sufficient to repress proliferation and promote differentiation genes expression a. RNA-sequencing identifies 321 significantly repressed (blue) and 595 significantly induced (red) genes in sg*PTPN11* iPNs compared to sgNTC iPNs. b. Gene ontology analysis shows genes induced in sg*PTPN11* iPNs are enriched for cell differentiation and negative regulators of cell proliferation.

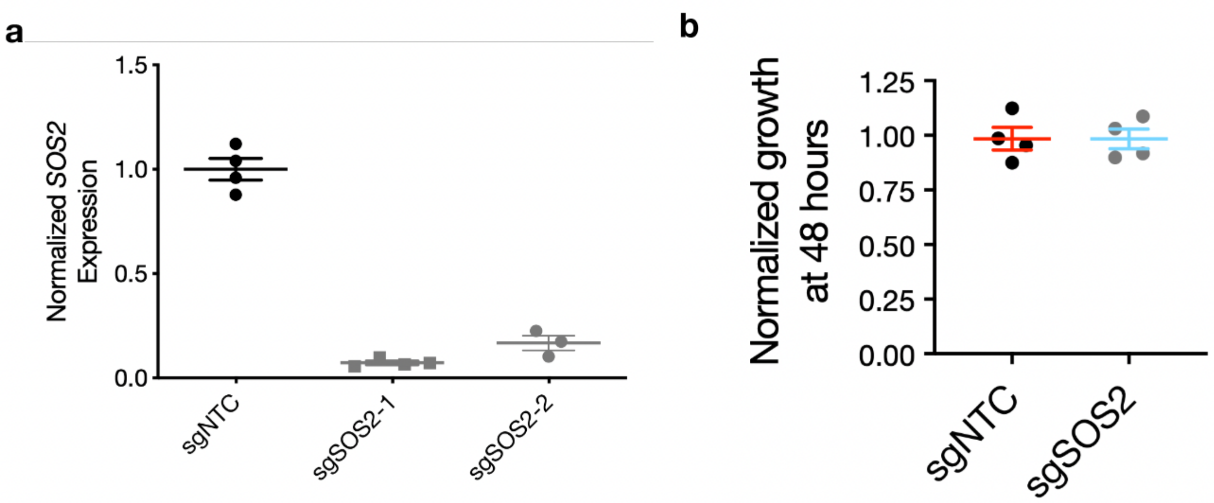

Figure S7. CRISPRi sg*SOS2* repression does not affect cell growth. a. Validation by qRT-PCR of sg*SOS2* repression in iPNs. b. No significant difference in growth rate is observed upon sg*SOS2* repression.

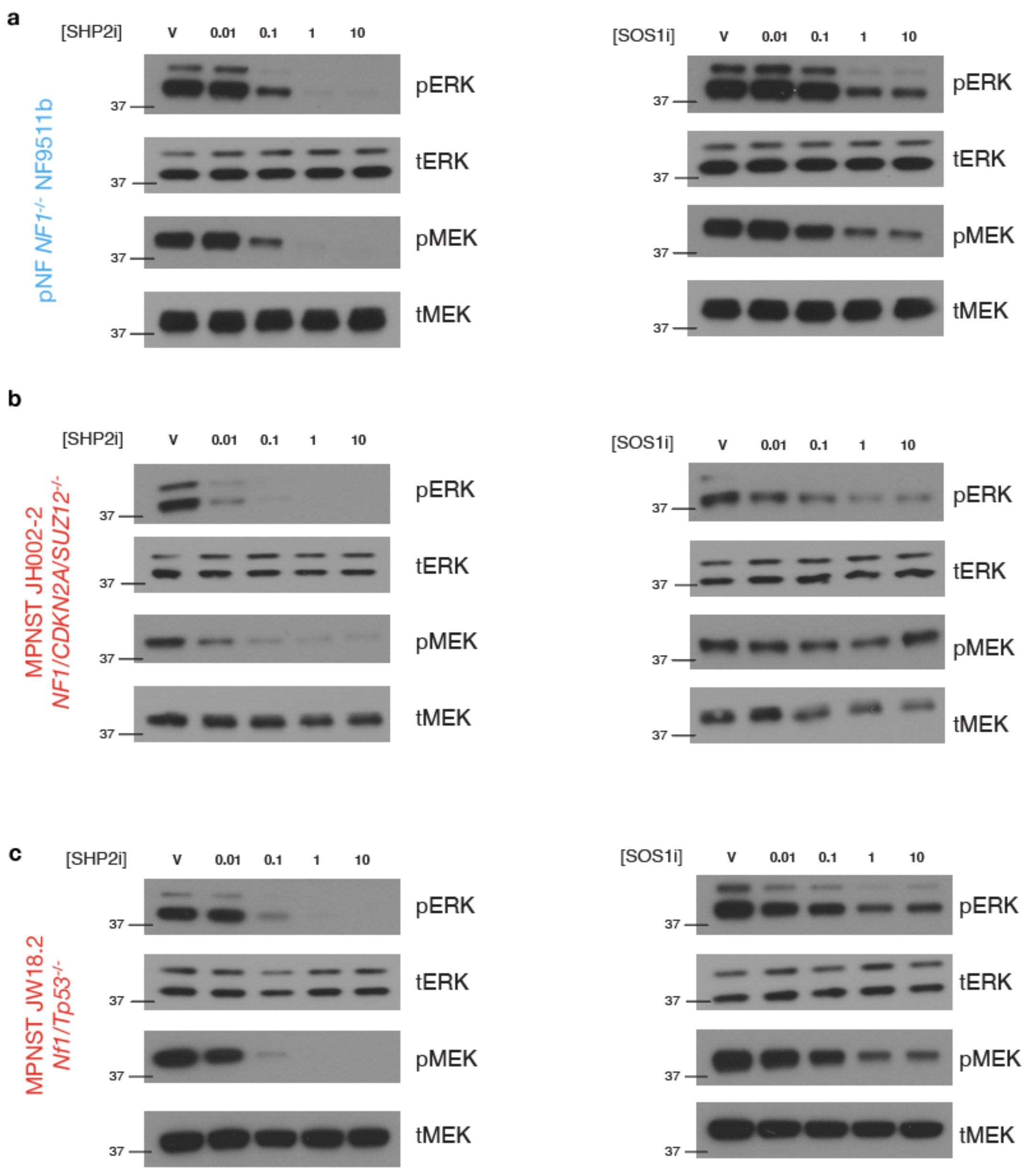

Figure S8. SHP2 inhibition shows greater biochemical repression across a panel of plexiform neurofibroma (pNF) NF9511b, MPNST JH002-2, and MPNST JW18.2 tumor cell lines. The SHP2 inhibitor RMC4550 shows greater efficacy than the SOS1 inhibitor BI-3406 in a. plexiform neurofibroma (pNF) NF9511b, b. malignant peripheral nerve sheath tumors (MPNST) JH002-2, and c. MPNST JW18.2 cells.

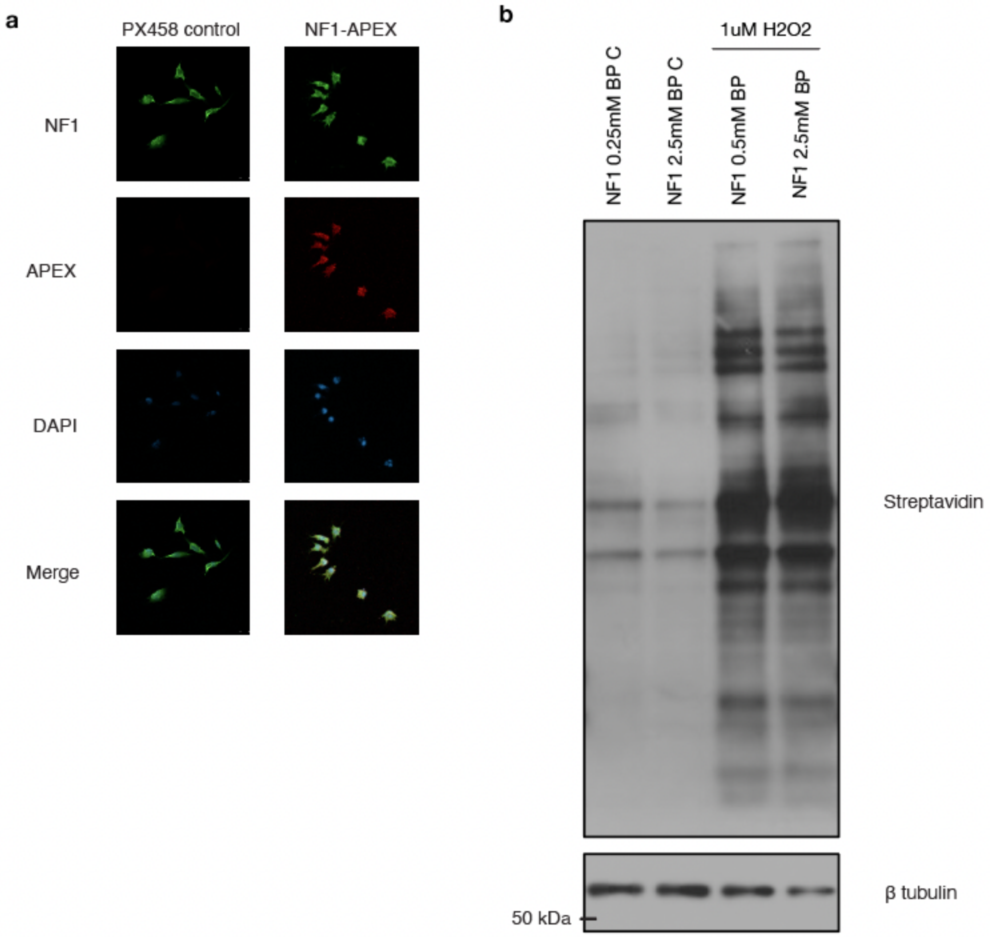

Figure S9. Validation of NF1-APEX system in iPN cells. a. Transient transfection of the NF1-APEX construct in iPN cells does not affect NF1 localization. b. Streptavidin immunoblot following biotin-phenol +/- hydrogen peroxide treatment confirms capture of the NF1 proximal proteome.

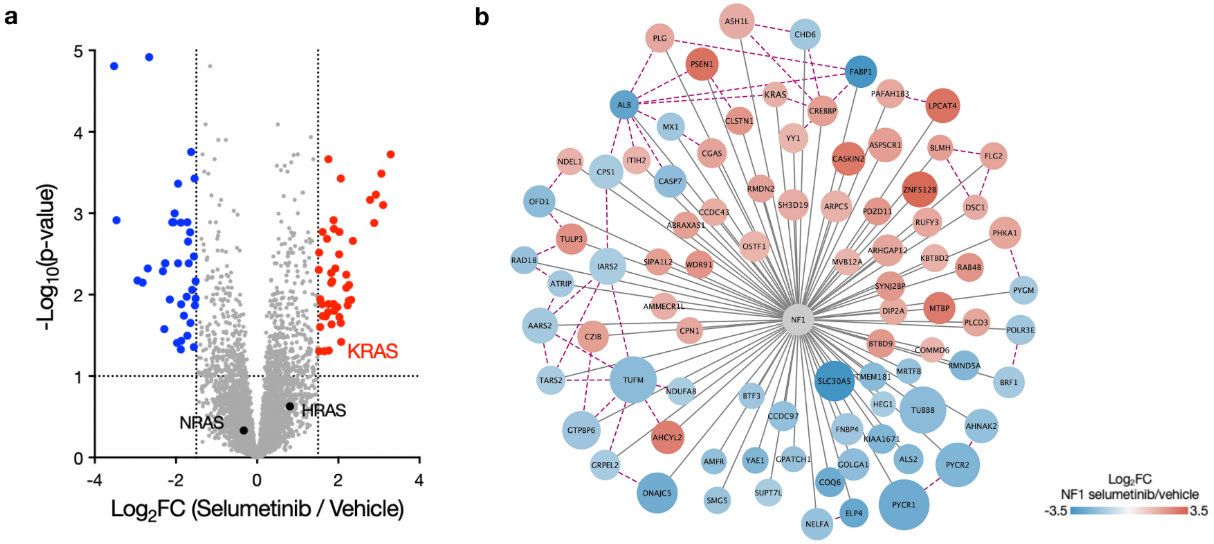

Figure S10. APEX proximal proteomic mass spectrometry nominates KRAS is the predominant Ras protein downstream of NF1 in the peripheral nervous system. a. Volcano plot and b. direct interaction network of pairwise differentially identified NF1 proximal proteins shows 44 significantly increased (red) including KRAS and 37 significantly decreased (blue) proteins following selumetinib treatment in iPNs. Solid grey edges represent APEX-derived protein interactions. Dotted purple edges represent STRING-derived functional associations. Size of node is proportional to $-\log_{10}$pvalue of selumetinib vs control.

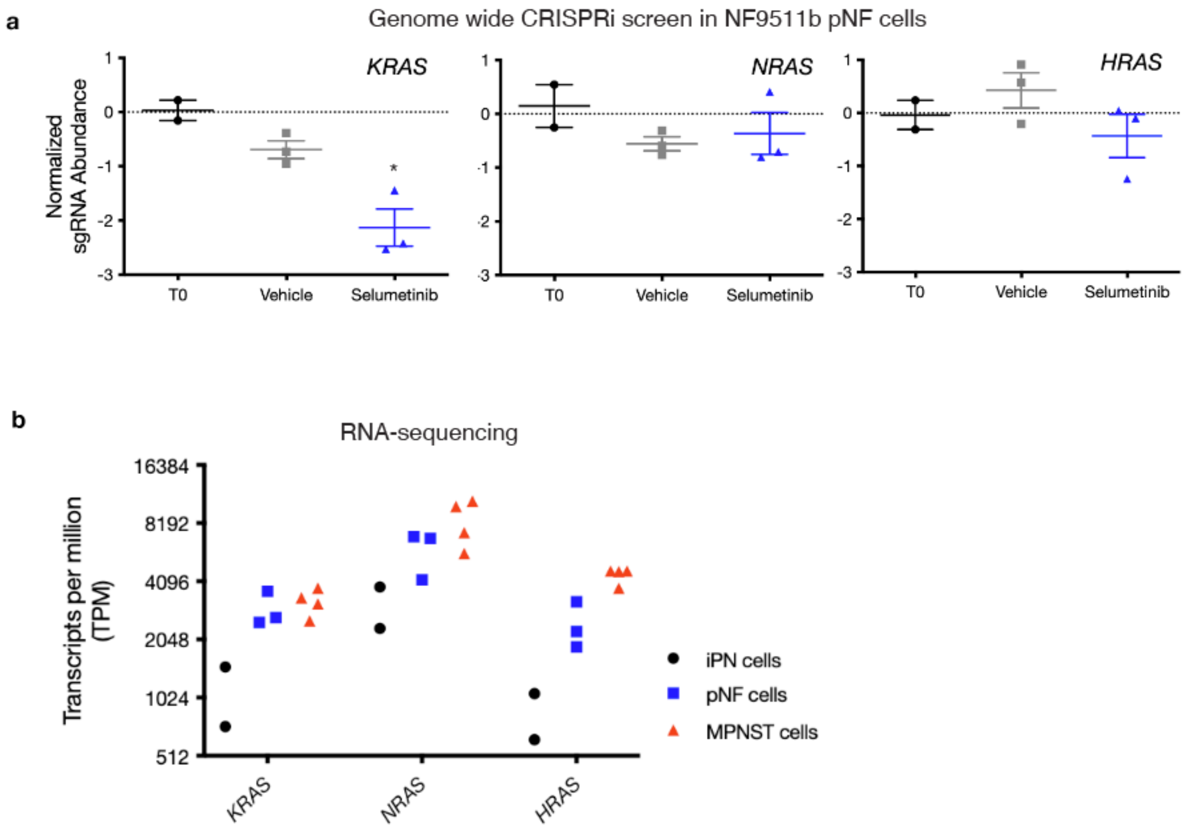

Figure S11. Functional genetic and pharmacologic approaches support a key role for KRAS in *NF1* mutant PNS cells. a. Analysis of published genome wide CRISPRi screens in *NF1* mutant pNF cells show *KRAS,* but not *HRAS* or *NRAS*, repression sensitizes pNF cells to the MEK inhibitor selumetinib.[6] b. All three classic Ras genes *(KRAS, NRAS, HRAS)* are expressed at the mRNA level in iPN, pNF, and MPSNT cells.

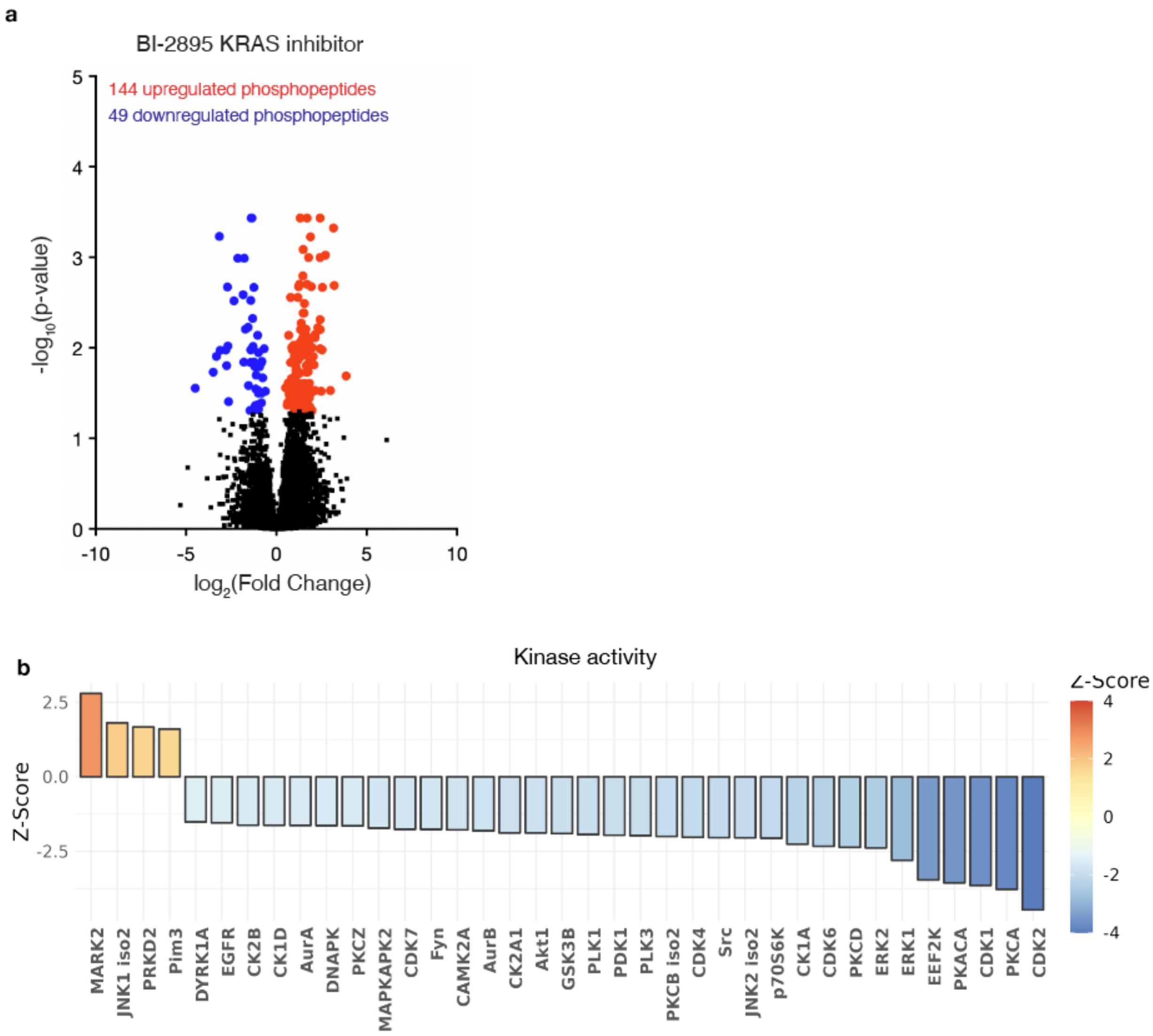

Figure S12. PTM MS analysis of *NF1* mutant NF9511b pNF cells treated with 1uM KRAS inhibitor BI-2865. a. A total of 144 significantly upregulated phosphopeptides and 49 significantly downregulated phosphopeptides ($p < 0.05$) were observed. b. ROKAi kinase enrichment analysis reveals multiple repressed kinases following KRAS inhibition including expected CDK1/2 and ERK1/2 downstream Ras effectors.

Supplementary Datasets.

Dataset S1. Normalized Gene Expression Values from RNA-sequencing of CRISPRi sgNTC, sgNF1, sgPTPN11, and sgSOS2 immortalized peripheral nerve (iPN) cell panel.

Dataset S2. Phosphopeptide abundances for significantly regulated peptides in CRISPRi sgNTC or sgNF1 cells treated with DMSO or selumetinib.

Dataset S3. Significant ROKA AI Kinase Activity Analysis Hits for PTMs by cluster following selumetinib treatment in sgNTC and sg*NF1* iPN cells.

Dataset S4. Cancer Dependency Map (DepMap) analysis of top 100 genes significantly correlated with NF1

Dataset S5. Significantly repressed or induced genes following CRISPRi sgPTPN11 or sgSOS2 repression in iPNs.

Dataset S6. NF1-APEX significantly modulated proximal proteins following selumetinib treatment in iPNs.

Dataset S7. Significant ROKA AI Kinase Activity Analysis Hits for PTMs following KRAS inhibitor BI-2865 treatment.
